# Determination by UHPLC‐QTOF‐HRMS of Phosphatidylethanol (PEth) in Dried Blood Spots: Method Validation and Practical Application of a Rising Alcohol Abuse Biomarker With Minimally Invasive Sampling

**DOI:** 10.1002/bmc.70081

**Published:** 2025-04-11

**Authors:** Christina Ververi, Marta Massano, Eugenio Alladio, Alberto Salomone, Marco Vincenti

**Affiliations:** ^1^ Department of Chemistry University of Turin Turin Italy; ^2^ Centro Regionale Antidoping Orbassano TO Italy

**Keywords:** alcohol abuse, DBS, dried blood spot, PEth, Q‐TOF

## Abstract

The goal of our study was to develop and validate a simple, quick, and sensitive method to detect phosphatidylethanol (PEth) in dried blood spots (DBS). A 30‐μL aliquot of blood was collected on a DBS card and allowed to dry at room temperature. Then, the spot was cut and transferred into a clean tube where the internal standard (PEth‐D5) and 1‐mL hexane were added followed by stirring, sonication, and centrifugation at room temperature. The dried supernatant was reconstituted with 30‐μL acetonitrile and analyzed by UHPLC‐HRMS‐QTOF. Calibration curve was created at 20, 50, 100, 200, 300, and 500 ng/mL; the limit of detection was calculated at 5 ng/mL (S/N > 3) while accuracy, precision, recovery, and matrix effect were successfully evaluated, along with the analyte stability at different time intervals and temperatures. The study demonstrates that quantifying PEth 16:0/18:1 from DBS cards is feasible using UHPLC‐QTOF or UHPLC‐QqQ instrumentation while the QTOF method was validated and proved reliable for PEth detection to assess both excessive alcohol consumption and alcohol abstinence, matching current guidelines. Preliminary data on authentic samples confirmed the method's performance in terms of ease, sustainability, and speed, supporting its great potential for routine toxicological diagnosis of chronic alcohol abuse.

## 1 Introduction

Alcohol is potentially a psychoactive and addiction‐inducing substance, whose abuse is widespread in Europe (WHO 2019). According to the World Health Organization (WHO), alcohol consumption has risen to unprecedented levels, resulting in a surge in accidents, crimes, and diseases linked to alcohol abuse and dependence (WHO 2019). Binge drinking contributes to car accidents and aggressive behaviors, while chronic excessive drinking leads to severe health issues such as liver cirrhosis, cardiovascular diseases, and cancers (Gmel and Rehm 2003; Rehm and Shield 2019). Consequently, accurate detection of chronic or frequent excessive alcohol intake is often required by authorities in clinical or forensic contexts, including workplace testing, driving license issuance, child custody decisions, organ transplantation, and alcohol withdrawal monitoring.

Phosphatidylethanol (PEth) is a group of abnormal phospholipids formed in the body only when alcohol is present. PEth has gained popularity as a biomarker for alcohol abuse because it accumulates in red blood cells, providing a detection window of 2–4 weeks (Helander and Zheng 2009; Luginbühl et al. 2022). The most abundant and commonly used PEth analog for detecting chronic or excessive alcohol intake is PEth 16:0/18:1. PEth formation peaks within 8 h of alcohol consumption and remains detectable for 3–4 weeks after stopping heavy drinking. Notably, PEth is not detectable in teetotalers (Beck et al. 2018; Nalesso et al. 2011). Due to its extended detection window, PEth serves as an alternative or supplement to ethyl glucuronide (EtG) in hair, the gold standard for chronic alcohol abuse detection (Marques et al. 2010; Morini et al. 2009; Pirro et al. 2011, 2013).

Dried blood spot (DBS) is a microsampling technique where capillary blood is collected on filter paper, typically from a finger prick, to minimize blood volume variation. The blood is dried under controlled conditions to prevent damage. This minimally invasive method uses small blood volumes, making it safe, quick, and easy to handle, with low impact on individuals. The collection and preanalytical process are simple and resistant to manipulation.

In 2011, Faller et al. demonstrated the feasibility of detecting PEth in both whole blood and DBS, showing good correlation between venous blood and DBS results (Faller et al. 2011). Since then, there have been significant advancements in sample preparation, handling, and analysis, including the automation of sample prep, which has proven effective in reducing analysis time (Fatch et al. 2022). However, automation is not yet practical for small sample batches, limiting its use in forensic laboratories that need routine PEth analysis.

The present study was aimed to develop and validate a sustainable and high‐performance laboratory method using ultra‐high‐pressure liquid‐chromatograph (UHPLC) coupled with a quadrupole time‐of‐flight (QTOF) tandem high resolution mass spectrometer (HRMS) to determine PEth 16:0/18:1 concentration in DBS samples and evaluate its applicability to the diagnosis of alcohol abuse in routine laboratory workflow. Then, the results were compared with those obtained with a triple quadrupole (QqQ) tandem mass analyzer working in the selected reaction monitoring (SRM) mode as the use of LC‐MS/MS is thought the gold‐standard for this kind of analysis (Faller et al. 2011; Fatch et al. 2022; Luginbühl et al. 2021; van Uytfanghe et al. 2021).

The method offers several advantages, including the use of only 30 μL of blood, a simple and fast protocol, and sustainability aligned with Green Chemistry principles, such as reduced solvent use, low waste, and minimal energy consumption. The HRMS‐QTOF system enhances sample throughput, with the SWATH acquisition method that captures all MS and MS/MS spectra of detectable ions in a single run, allowing for future questions to be addressed without rerunning samples. This results in lower analysis costs, reduced sample turnaround time, fewer instruments needed, and a streamlined laboratory workflow.

## 2 Materials and Methods

### 2.1 Reagents and Preparation of Standards

Methanol, isopropanol, ammonium acetate, formic acid, hexane, and acetonitrile, were purchased from Sigma‐Aldrich (Milan, Italy). The analytical standard of PEth 16:0/18:1, PEth‐D5, and the WHATMAN FTA DMPK C cards were also purchased from Sigma‐Aldrich (Milan, Italy). Ultra‐pure water was obtained from a Milli‐Q UF‐Plus apparatus (Millipore, Bedford, MA, USA). Stock standard solutions were prepared in methanol at 1 mg/mL and stored at −20°C until used. Working solution of PEth 16:0/18:1 and the internal standard (ISTD) PEth‐D5 solution were prepared at the final concentration of 1 μg/mL by dilution with methanol.

### 2.2 Sample Preparation

The spiked specimens used in the method development and validation were prepared from a blank whole blood matrix obtained by mixing aliquots collected from 5 abstainer volunteers, stored in EDTA blood collection tubes at 4°C. Matrix effect was tested using the five separate specimen, each spiked with the relevant volume of PEth working solution. Ethical consent for the project “Diagnosis and Biomonitoring by means of Dried Blood Spots micro sampling systems” was obtained from the Bioethical Committee of the University of Turin (prot.n. 0459057/2024). The samples were tested with the QqQ for the presence of PEth and found to be negative; 50‐μL aliquots of blank blood were fortified at six concentration levels (20, 50, 100, 200, 300, and 500 ng/mL) with PEth 16:0/18:1. Then, 30 μL was spotted on a WHATMAN FTA DMPK C card using a calibrated pipette after the blood sample was vigorously stirred. The sample preparation has been performed volumetrically so there is no inference by hematocrit level. At this level the hematocrit phenomenon was not evaluated. For future large clinical studies, the hematocrit range of the calibrators should be determined and matched to the one of the target populations. The spot was allowed to dry for at least 3 h at room temperature, away from direct sunlight. The entire spot on the DBS card was punched out and transferred into a glass tube in which the following solutions were consecutively added: 250‐μL water:isopropanol:2‐mM ammonium acetate with 0.01% formic acid (2:4:0.2 vol/vol/vol), 1.5‐μL internal standard PEth‐D5 (1 μg/mL) (final concentration with respect to the blood content 50 ng/mL), and 1‐mL hexane as the extraction solvent. The tube underwent intense stirring and ultrasonication for 30 min at room temperature followed by 10 min in a multimixer and 10‐min centrifugation at 13,000 g. The supernatant was transferred into a fresh tube prior to evaporating the solvent under a nitrogen flow at room temperature (approximately 21°C). The dry residue was reconstituted with 30‐μL acetonitrile centrifuged for 10 min at 4000 rpm and either 5 or 2 μL of the resulting solution—depending on the instrument—was injected into the UHPLC system.

### 2.3 Instrumentation

**FIGURE 1:**
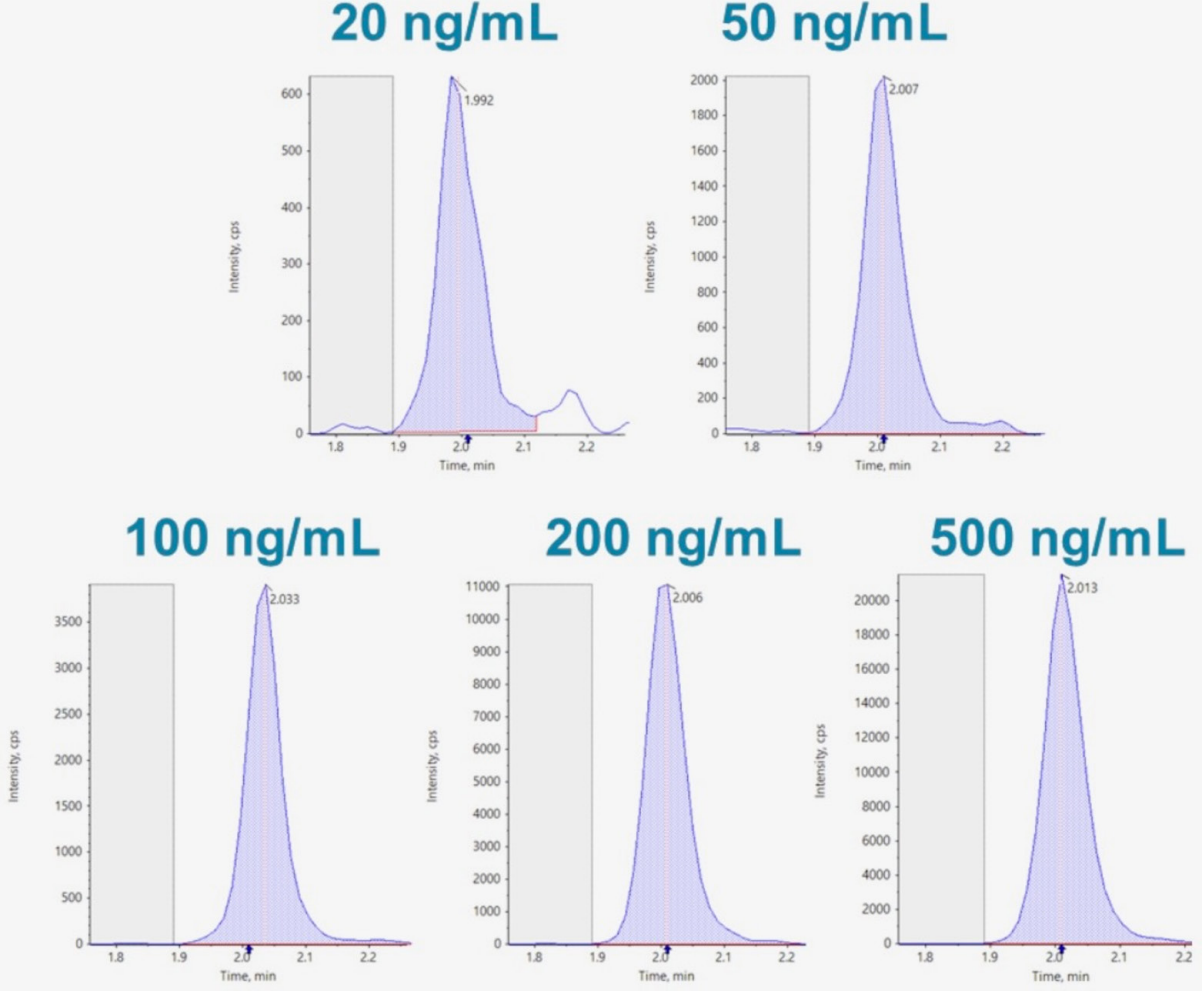
Characteristic chromatograms of PEth 16:0/18:1 at concentrations ranging from 20 to 500 ng/mL obtained with the use of SWATH on the SCIEX X500R QTOF.

The method development and validation were conducted using a UHPLC SCIEX ExionLC AC (AB SCIEX, Framingham, USA) coupled with a quadrupole/time‐of‐flight SCIEX X500R (QTOF) mass spectrometer equipped with a Turbo VTM ion source operating in electrospray negative‐ion mode (SCIEX, Darmstadt, Germany). In the QTOF instrument, a preliminary high‐resolution full scan spectrum was acquired, followed by a MS/HRMS product ion spectrum collection. The advantage of the SWATH acquisition method is that a single generic MS acquisition method is used, meaning that the mass spectrometer does not require the initial detection of an MS peak to proceed to MS/MS analysis, but it uses a wider Q1 isolation window and steps it across the entire m/z mass detection range, thus providing the possibility to get the full MS and MS/MS picture of every detectable peak in each sample without the need to reinject it. The qualitative identification was based on the accordance of the retention time, precursor ion, and characteristic fragment ions m/z values (the latter in high resolution with an accepted mass error < 5 ppm) with the pure PEth 16:0/18:1 and its theoretical exact mass values. Table 1 summarizes all the source and gas parameters, as well as the TOF MS and TOF MS/MS parameters used at the acquisition. Figure 1 shows characteristic chromatograms of PEth 16:0/18:1 at concentrations ranging from 20 to 500 ng/mL obtained with the use of SWATH on the SCIEX X500R QTOF.

**TABLE 1:** QTOF source and gas parameters, TOF MS, and TOF MS/MS parameters.

	MS method	Peth SWATH
	Method duration	10 min
	Polarity	Negative
	Spray voltage	−3500 V
Source and gas parameters	Ion source gas 1	30 psi
	Ion source gas 2	40 psi
	Curtain gas	25 psi
	CAD gas	7 psi
	Temperature	450°C
TOF MS	TOF start mass	124 Da
	TOF stop mass	793 Da
	Accumulation time	0.05 s
	Declustering potential	−61 V
	DP spread	0 V
	Collision energy	−10 V
	ce spread	0 V
TOF MS/MS	TOF start mass	60 Da
	TOF stop mass	707 Da
	Accumulation time	0.05 s
	Declustering potential	−61 V
	DP spread	0 V
	Collision energy	−10 V
	ce spread	0 V

The results obtained for some crucial features of the method were compared with the ones obtained with a UHPLC SCIEX ExionLCTM AC (AB SCIEX, Framingham, USA) coupled to a SCIEX Triple Quad 4500 system (QqQ) (Sciex, Darmstadt, Germany). For the QqQ instrument, a SRM acquisition mode was used, and the qualitative identification was based on the same principles as above, except that the product ions were selected in low resolution. The SRM transitions used to determine PEth were 701.5 → 281.0, 701.5 → 254.9, 701.5 → 437.3, and 706.5 → 281.0 for PEth‐D5.

Concerning the instrumental analysis characteristics, both UHPLC were equipped with a Waters ACQUITY UPLC BEH HILIC column (100 × 2.1 mm, 1.7 μm) maintained at 40°C. The mobile phases for both chromatographic programs consisted of formic acid 0.1% and ammonium formate 10 mM in water (A) and acetonitrile (B). The flow rate was set at 0.5 mL/min. The mobile phase composition was programmed as follows: Elution started from A:B (v:v) 5:95 and retained for 1 min, elution at 30:70 until 3.5 min, and final re‐equilibration for 1.5 min to restore the initial conditions. The total run time was 5 min for both programs. HILIC stationary phase was chosen as it retains polar, hydrophilic compounds that are not retained on standard reversed phase columns and, complementary to strong reversed phase elution solvents starting at high organic percentage, can elute polar compounds like PEth, which is highly lipophilic. Moreover, the HILIC mode column simplifies the sample preparation for metabolites, like PEth, because the metabolic process generally results in the addition of polar groups to enhance elimination from the cellular tissue. Lastly, the injection volume was 5 μL for the QTOF and 2 μL for the QqQ instruments, respectively. The difference in the injection volume stands for the difference in the sensitivity of the two instruments. The triple–quadrupole instrument is known for its sensitivity, and it is used as the gold standard for quantification and quantification of such analytes at low concentration levels. The QTOF offers other advantages that cost a percentage of sensitivity, thus leading to different injection volumes.

### 2.4 Authentic Samples

For all the samples analyzed, ethical consent for the project “Diagnosis and Biomonitoring by means of Dried Blood Spots micro sampling systems” was obtained from the Bioethical Committee of the University of Turin (prot.n. 0459057/2024). The samples were tested with the QqQ for the presence of PEth and found to be negative.

Preliminary experiments were performed to evaluate the method's performance in authentic blood samples; 20 whole blood samples, collected in EDTA blood collection tubes at 4°C until spotted, deriving from driving relicense program for routine analysis were spotted (30 μL) onto WHATMAN FTA DMPK C filter papers. The aforementioned extraction protocol was applied, and the PEth concentration was determined. Following, 17 DBS samples were collected and analyzed at the laboratory as a part of the research study to further confirm the method's performance in daily practice. DBS collection was approved by the Ethics Committee of the hospital “SAN LUIGI GONZAGA,” and the volunteers participated after signing a written consent. For the method testing, samples were collected from persons in a driving license program, while no other patient‐related information was disclosed as ethical protocol ensures. The sample collection was performed by simply pricking the finger and then depositing the blood drop onto a volumetric CapitainerB Vanadate collection card. The instructions of the manufacturer were followed as described at their brochure.^1^

The use of CapitainerB Vanadate was selected to also examine and demonstrate the applicability of the method as an on‐site solution for alcohol determination. Before application, a preliminary experiment comparing the WHATMAN FTA DMPK C cards with the CapitainerB Vanadate collection card has been executed and no significant difference was noticed while full comparison of DBS cards with volumetric DBS card has been performed by Beck et al. (2018). Nevertheless, CapitainerB Vanadate card was preferred because it is especially designed for the detection of PEth and the enhancement of the stability. The hematocrit phenomenon did not pose any inference as the collection cards are hematocrit independent designed and the exact amount of blood is being advanced via microfluidic tube. Moreover, the correlation of values of PEth obtained by capillary and venous blood has been already extensively demonstrated and there is no significant difference between them (Beck et al. 2018; Faller et al. 2011; Kummer et al. 2016). Then, the samples were stored at approximately 23°C prior to the analysis, and they were analyzed 1–3 days after sampling, following the extraction protocol described above.

## 3 Method Validation

For the method validation, spiked samples were prepared and analyzed in three working sessions along 5 days based on previously published protocols for the validation of analytical methods (Alladio et al. 2020a, 2020b). The performance parameters evaluated with this data set included calibration curve, intraday and interday accuracy and precision, limit of detection (LOD), and limit of quantification (LOQ). Recovery, matrix effect, and analyte stability were investigated by executing further independent experiments. Several of these samples were reanalyzed by the QqQ instrument, and the results were evaluated and used to confirm the analytical method's performance on a commercial instrument other than the QTOF at concentration levels (20, 100, 200, and 300 ng/mL) close to the cut‐off values. The method used in QqQ was validated to evaluate linearity and accuracy (data not shown).

### 3.1 Calibration

The calibration curve was created by calculating the peak‐area ratios between the target analyte and the internal standard and then plotting them on the *y*‐axis against the known concentration levels (*x*‐axis). The homoscedastic or heteroscedastic distribution of the data was evaluated by examining the variance of nine data points at six concentration levels; by testing the variance increase from low to high concentrations (*p* < 0.05), heteroscedasticity was recognized, and the relative weighting factor was assessed (1/*x* or 1/*x* ^2^) depending on the linear or quadratic relationship occurring between concentration and variance (Gu et al. 2014). The order of the calibration model (linear vs. quadratic) was chosen on the basis of Mandel and lack‐of‐fit test results (Gu et al. 2014).

### 3.2 LOD and LOQ

For the calculation of LOD and LOQ, the Hubaux–Vos method was used considering the three lowest calibration points and 90% confidence intervals, corresponding to CCα = CCβ = 5% errors. The calculated LOD value was tested further with independent experiments on intentionally spiked samples at the predicted LOD level and then decreasing the spiked concentration until a signal‐to‐noise (S/N) ratio lower than 3 was observed. The final accepted LOD value corresponded to an S/N higher than 3 (Hubauxl and Vos 1970). LOQs were attributed to the lowest concentration of the calibration range yielding accuracy values lower than the accepted limit.

### 3.3 Accuracy and Precision

Intraday and interday accuracy (expressed as bias%) were also calculated for each of the 3 days based on Alladio et al. (2020a).

Taking into account the variability factors affecting the sampling operations and the tiny blood volume collected, the accuracy was considered optimal if the bias was lower than 15% and good with bias < 20%. The method was deemed as validated if the average intraday and interday accuracy over all calibration levels remained below 20% even if single values randomly exceeded ± 20% (Alladio et al. 2020a).

Intraday and interday precision, expressed as coefficient of variation (CV%), was independently assessed for the 3 days of analysis. In practice, the protocol used for calculating accuracy and precision is based on the same data collected for preparing the calibration curve, obtained in the three separate days (Massano et al. 2022).

### 3.4 Matrix Effect and Extraction Recovery

The matrix effect was estimated at the concentration level of 20 ng/mL by comparing the experimental results obtained from three blank whole blood samples and three hexane solutions, all equally spiked (with target analyte and ISTD) after the extraction step. The ionization suppression/enhancement was expressed as the mean percentage ratio between the two measured signals (Scientific Working Group for Forensic Toxicology 2013).

The extraction recovery was determined at the concentration level of 500 ng/mL, by comparing the experimental results obtained from six whole blood samples, three of which spiked before and three after the extraction step. The result was expressed as the mean percentage ratio between the two signals completed with its uncertainty, expressed as extraction repeatability (CV%).

### 3.5 Stability

The stability of the target analyte was examined on both purposely spiked blank samples and real blood samples along different temperatures and time intervals. The reasoning of the stability tests is to evaluate at first level the stability of the analyte onto the DBS card when storing for shorter or longer periods of time compared with the initial one in the context of a forensic toxicology laboratory that stores the samples for confirmation analysis. The conditions selected stimulated the possible realistic scenarios concerning transportation (e.g., from the sampling site to laboratory) and short/long‐term storage (in case of confirmation analysis).

Spiked samples were spiked based on the aforementioned protocol at 20, 200, and 500 ng/mL (three replicates each, for each day, for both temperatures), left to dry at room temperature (RT = approximately 21°C) and at 4°C (away from direct sunlight), and stored before analysis for 1, 3, 7, and 15 days. Globally, 72 DBS samples were analyzed. The concentration levels of 20 and 200 ng/mL were selected because they are suggested as PEth 16:0/18:1 cut‐off limit for the ascertainment of abstinence and excessive alcohol intake respectively, by the consensus document recently published by the *PEth‐NET society* (Luginbühl et al. 2022). The concentration level of 500 ng/mL was selected as suggestive of definite alcohol abuse. Likewise, whole blood received from five volunteers and was prepared likewise. The stability conditions were observed through raincloud plots that traces the concentrations calculated at the different storage temperatures and after different storage intervals (Capiau et al. 2019) in comparison with those measured immediately.

## 4 Results and Discussion

### 4.1 Calibration

The present method proved capable of detecting the target analyte at all concentration levels tested. Residues analysis and variance distributions at low, medium, and high concentration levels indicated strong heteroscedasticity of data points and quadratic dependence of the variance on PEth concentration, suggesting the adoption of a 1/*x* ^2^ weighting factor for the calibration model. The resulting equation was as follows: *y* = −0.1098 + 0.6025*x* − 0.0092*x* ^2^. To verify the calibration model further, a linear regression F tests was performed, showing statistical significance of the quadratic component with a *p* value lower than 0.05.

### 4.2 LOD and LOQ

A LOD value of 9.7 ng/mL was calculated by the Hubaux–Vos algorithm, (Hubauxl and Vos 1970). As described in Section 3.2, the calculated LOD values were tested further with independent experiments to verify minimum level still yielding a S/N ratio higher than 3 (Capiau et al. 2019). A LOD of 5 ng/mL was experimentally obtained, confirming that the Hubaux–Vos method provides a conservative estimation of LOD, in agreement with the high confidence limits adopted. Similar or lower LOD values for PEth were obtained after intravenous blood sampling at the expense of substantially more complex sample storage and preliminary treatment procedures (Casati et al. 2019; Müller and Føreid 2022; White et al. 2022). The LOQ value was attributed to the lowest concentration of the calibration range, which provides adequate accuracy; consequently, the assigned LOQ for the present determination is equal to 20 ng/mL.

### 4.3 Accuracy and Precision

Interday and intraday accuracy and precision for the six concentrations tested are reported in Table 2. The intraday and interday accuracy obtained from the QTOF proved optimal for almost all the calibration levels with bias% values lying below 15%; only at 50 ng/mL was this limit exceeded, possibly due to overlapping sources of random variability. The intraday and interday precision were within ± 20% in the 100–500 ng/mL interval (i.e., that useful for the assessment of excessive alcohol consumption) and moderately higher for the lowest points of calibration range. These results were compared with those obtained at the key‐point concentrations (20 and 200 ng/mL) from the QqQ instrumentation and proved consistent with them (CV% and bias % < 15%) (Table 2).

**TABLE 2:** Intraday and interday accuracy and precision expressed as Bias% and CV% for QTOF (six levels) and QqQ (two levels) instrumentations.

PEth 16:0/18:1 (ng/mL)	ACCURACY (Bias%)				PRECISION (CV%)			
	Intraday QTOF	Intraday QqQ	Interday QTOF	Interday QqQ	Intraday QTOF	Intraday QqQ	Interday QTOF	Interday QqQ
20	12.3	−0.6	−15.6	−19.8	34.6	32.6	25.8	14.7
50	−20.4		−27.4		23.4		28.0	
100	9.9		8.7		8.4		8.2	
200	15.0	4.3	14.9	4.8	11.4	13.1	11.7	30.0
300	6.2		3.7		15.6		15.6	
500	3.4		−8.6		20.0		19.7	

The relatively worse results obtained at 20 and 50 ng/mL have significance only if PEth determination is used to assess alcohol abstinence, not for the detection of alcohol abuse. From literature data and our own preliminary experiments on real samples, even a moderate drinker has significantly higher PEth concentrations (> 50 ng/mL), while the cut‐off proposed for excessive alcohol intake is higher than 200 ng/mL (Luginbühl et al. 2021, 2022; Marques et al. 2010).

### 4.4 Matrix Effect and Extraction Recovery

The extraction recovery obtained from triplicated experiments was 34 ± 5%. The relatively low extraction recovery is expected and is coherent with previous studies using DBS for the determination of other analytes (Massano et al. 2022; van Uytfanghe et al. 2021), because the DBS procedure involves extraction of a dried substrate from a relatively hydrophilic support, leading to potential interactions with the endogenous components present in the DBS matrix (Koster et al. 2015). The matrix effect substantially indicates absent or limited ion suppression, corresponding to experimental values of −14% and −3% recorded from QTOF and QqQ instrumentation, respectively.

### 4.5 Stability

**FIGURE 2:**
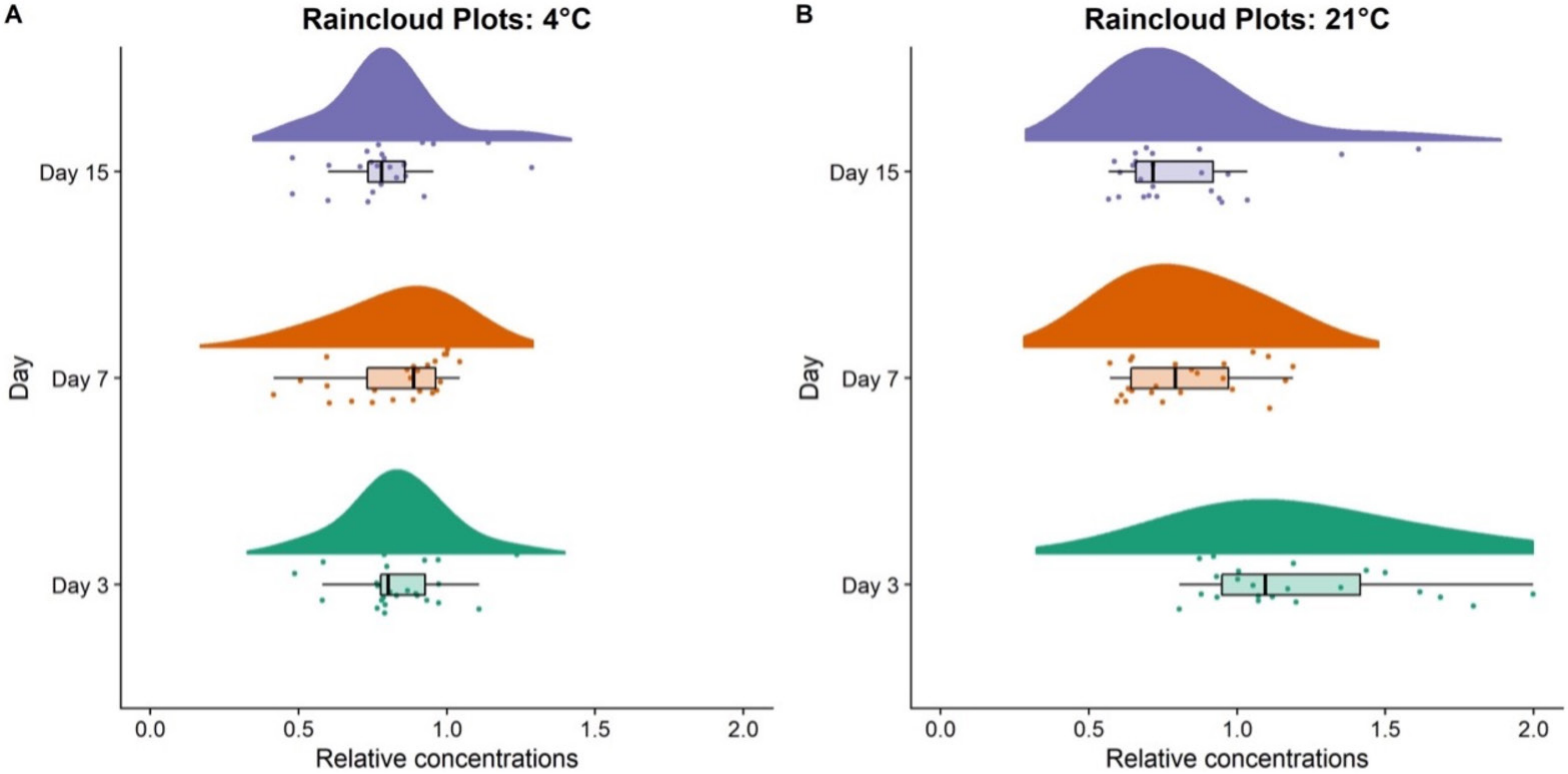
Relative concentrations of PEth 16:0/18:1 after 3, 7, and 15 days of storage at 4°C (a) and 21°C (b), with respect to the concentrations recorded at Day 1.

The stability results are comprehensively described by the raincloud and box‐and‐whiskers plots reported in Figure 2a,b (at ambient temperature and 4°C) showing the ratio between the recorded concentration after 3, 7, and 15 days of storage, with respect to the concentrations recorded at Day 1.

At both room temperature and 4°C, the data point distributions and median showed a slight decreasing trend due to PEth degradation, which literature data report to start after 3–5 days (Heier et al. 2016). This degradation trend is apparently less pronounced when the samples are stored in refrigerated conditions. These results are in accordance with the literature findings that compared the PEth stability on DBS cards under similar conditions (Bakhireva et al. 2016). Overall, the observed ratio distributions show a maximum close to 1 in all the plots, demonstrating limited PEth degradation even after 1–2 weeks, independently on the storage temperature. The proven PEth stability on DBS cards under different storage conditions and time intervals, combined with the ease of sampling and the cost‐effective transportation and storage protocols, provide the accountable authorities with a practical investigation tool and reliable analysis even when the sampling place is remote and several days necessarily elapse from sampling to laboratory delivery.

### 4.6 Clinical Samples

After validation, preliminary experiments were performed to evaluate the method's performance in 20 authentic blood samples that were spotted 20 whole blood samples, collected in EDTA blood collection tubes at 4°C until spotted, deriving from driving relicense program for routine analysis (30 μL) onto the filter paper for the identification and quantification of the target analyte. From the 20 samples, five samples were found positive with concentrations ranging from 20 to 500 ng/mL and three to concentrations over 500 ng/mL. Four samples were found below LOQ and eight below LOD.

After having confirmed the applicability of the method, to further test it as an on‐site application, the DBS samples collected with CapitainerB Vanadate devices were analyzed. From the 17 DBS samples collected from volunteers, only two tested positive to PEth, with concentrations of 37 and 66 ng/mL, respectively. The other samples showed no significant PEth signal (below the LOD).

## 5 Conclusion

The study demonstrates that PEth 16:0/18:1 detection and quantification using minimal blood volume on DBS cards is reliable with UHPLC‐Q‐TOF. The validated QTOF method offers potential for routine and research use in assessing excessive alcohol consumption and alcohol abstinence, with cutoff concentrations for abstinence (< 20 ng/mL) and excessive consumption (> 200 ng/mL) within the measurable PEth range. While the stability of PEth on DBS cards has been confirmed, further research is needed to fully understand the toxicological significance of PEth levels and to explore the correlation between ethanol intake, metabolism, and PEth concentration.

DBS cards are ideal for PEth analysis due to their minimally invasive nature, easy sample collection, and long‐term PEth stability, even at ambient temperature. The method aligns with Green Analytical Chemistry principles, using minimal blood volume and reducing waste. QTOF‐HRMS instrumentation, particularly with SWATH acquisition mode, offers additional advantages by providing full MS and MS/MS spectra, enabling the detection of other phospholipids and facilitating re‐evaluation in contested cases. This approach allows for efficient, reliable, and cost‐effective alcohol monitoring worldwide.

Future research in PEth analysis should focus on several key areas to enhance its application and reliability. First, large‐scale studies are needed to explore the toxicological significance of PEth concentrations and their correlation with ethanol intake and metabolism, refining cutoffs for clinical and forensic use. Comparing PEth from DBS with other alcohol biomarkers, such as hair EtG, could expand the detection window and provide a more comprehensive approach to monitoring alcohol consumption and abstinence.

Further refinement of PEth cutoff values is essential, particularly for distinguishing moderate from heavy drinking, using real‐world sample data. Automating sample processing could reduce time and costs, improving efficiency for routine analyses, although challenges may remain for smaller forensic sample volumes. Expanding DBS use globally, particularly in low‐resource settings, could revolutionize alcohol monitoring, aided by the development of portable, cost‐effective systems for sample analysis and transport. Lastly, investigating the long‐term stability of PEth on DBS cards under different environmental conditions is also important to ensure the method's sustainability. Addressing these areas will lead to more refined methodologies, broader accessibility, and more accurate assessments of alcohol use and abstinence.

## Endnotes

^1^

https://capitainer.se/wp‐content/uploads/2022/08/DLB21‐001‐03D_CAPITAINER‐B‐VANADATE_IFU_EN.pdf.
